# DACH1 inhibits breast cancer cell invasion and metastasis by down-regulating the transcription of matrix metalloproteinase 9

**DOI:** 10.1038/s41420-021-00733-4

**Published:** 2021-11-12

**Authors:** Sattout Aman, Yanan Li, Yunmeng Cheng, Yuxi Yang, Linlin Lv, Bowen Li, Kangkai Xia, Shujing Li, Huijian Wu

**Affiliations:** 1 School of Bioengineering & Key Laboratory of Protein Modification and Disease, Liaoning Province, Dalian University of Technology, Dalian, China; 2 Present Address: 2 Ling Gong Road, Dalian, 116024 Liaoning China

**Keywords:** Cell invasion, Breast cancer

## Abstract

Human Dachshund homolog 1 (DACH1) is usually defined as a tumor suppressor, which plays an influential role in tumor growth and metastasis in a variety of cancer cells. However, the underlying mechanisms in these process are not yet fully clarified. In this study, DACH1 inhibited the invasion and metastasis of breast cancer cells by decreasing MMP9 expression. Mechanistically, DACH1 represses the transcriptional level of *MMP9* by interacting with p65 and c-Jun at the NF-κB and AP-1 binding sites in *MMP9* promoter respectively, and the association of DACH1 and p65 promote the recruitment of HDAC1 to the NF-κB binding site in *MMP9* promoter, resulting in the reduction of the acetylation level and the transcriptional activity of p65. Accordingly, the level of MMP9 was decreased. In conclusion, we found a new mechanism that DACH1 could inhibit the metastasis of breast cancer cells by inhibiting the expression of MMP9.

## Introduction

Breast cancer is the most common gynecological tumor, and for women, the incidence rate of breast cancer is the highest among multiple tumors [1]. Since there has been no effective treatment strategy for metastatic breast cancer, the tumor metastasis usually leads to high mortality in breast cancer patients [2]. Generally, the extracellular matrix (ECM) is often recognized as a non-cellular component of tissue, which can provide necessary biochemical and structural support for its cellular constituents [3]. Metastasis is a complex biological process, through which carcinoma in situ is transferred to the remote locations in the body. During the metastasis, the degradation of the basement membrane and the ECM are two important events [3–6]. Matrix metalloproteinase 9 (MMP9), also known as gelatinase B, together with MMP2 belong to the gelatinase subgroup of MMPs family, [7]. MMP9 plays an essential role in the generation and development of tumors. As a collagenase, MMP9 can degrade ECM, including type 4 collagen (an important component of basement membrane), thereby promoting the invasion and metastasis of tumor cells [7–9]. The expression of MMP9 is mainly regulated at transcription level. The expression of *MMP9* is largely determined by the proximal 670 bp of the *MMP9* promoter, and the 670 bp region contains the binding sites of AP-1, NF-κB, SP-1 Ets-1, etc [10, 11]. Therefore, the study on the transcription of *MMP9* may provide a possibility for revealing the fine regulation of MMP9 and finding a novel therapeutic target for breast cancer.

*MMP9* can be regulated by NF-κΒ signaling pathway via the NF-κΒ binding site in the *MMP9* promoter. The NF-κΒ family consists of p50 (NF-κB1), p52 (NF-κB2), p65 (RelA), c-Rel, and RelB. In the canonical NF-κΒ signaling pathway, p65/p50 complex binds to the NF-κB binding site in the promoter of target gene, leading to the transcriptional activation of the target gene [12, 13]. p300/CBP is the most important acetyltransferase of p65, and the acetylation of p65 is usually associated with the activation of NF-κΒ signaling pathways [14].

The cell-fate determination factor DACH1 is made up of 706 amino acids, containing Box-N and Box-C domains [15]. The expression of DACH1 is deficient or down-regulated in multiple carcinomas, suggesting that DACH1 might be a new tumor suppressor [16–18]. DACH1 exerts the repressive effects in the tumorgenesis by interacting with several proteins, such as p53 [19], YB-1 [20], SMAD4 [21], and c-Jun [22]. Specifically, DACH1 can also bind to NF-κB or AP-1 binding sites in the promoter of the target genes, which then inhibit the transcription of the target genes [16, 22, 23]. The previous work have shown that NF-κB and AP-1 bind sites are localized in the *MMP9* promoter, so we speculate that DACH1 may regulate the expression of *MMP9* through the two sites in the *MMP9* promoter.

In this study, DACH1 suppressed the invasiveness and metastasis of breast cancer cells via decreasing the expression of *MMP9*. DACH1 could interact with p65 and c-Jun respectively, and the ChIP-ReChIP assay showed that DACH1-p65 and DACH1-c-Jun complex could be recruited to the NF-κB binding site and AP-1 binding site respectively. Further study showed that DACH1 could promote the interaction between p65 and HDAC1, which then led to repressing the acetylation and transcriptional activity of p65.

## Results

### DACH1 inhibits migration and invasion of breast cancer cells

**Fig. 1:**
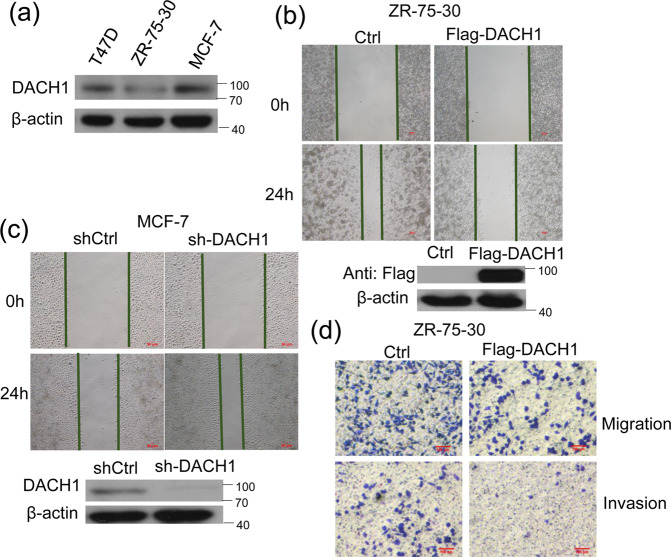
The effect of DACH1 on breast cancer cell migration and invasion. **a** The protein abundance of DACH1 was examined by western blot in T47D, ZR-75-30, MCF-7 cell lines. **b**, **c** Scratch wound-healing assay assessing the effects of DACH1 overexpression or knockdown on the motility of ZR-75-30 and MCF-7 cells. **d** Transwell migration and invasion assays evaluating the effects of DACH1 on the motility and invasion of ZR-75-30 cells. Cell migration and invasion assays were performed in 24-well chambers without or with Matrigel. Cells were transfected with Flag-DACH1 or control vector and then inoculated into the upper chamber. After cultured 24 h, cells on the lower surface of the filter were stained and photographed. The scale bars stand for 100 μm. Experiments were performed in triplicates and the data are representative of three independent experiments.

The expression of DACH1 in different breast cancer cell lines was examined by western blot analysis. The protein level of DACH1 was lower in ZR-75-30 cells than that in the T47D and MCF7 cells (Fig. 1a). The effect of DACH1 on the metastasis was evaluated via Scratch wound healing (Fig. 1b, c) and transwell migration assays (Fig. 1d). DACH1 overexpression reduced the migration ability of ZR-75-30 cells, while knockdown of endogenous DACH1 facilitated the migration of MCF-7 cells. Moreover, the Transwell invasion assay revealed that overexpression of DACH1 also inhibited the migration and invasiveness of ZR-75-30 cells (Fig. 1d). Our previous research has shown that DACH1 had no discernible influence on cell proliferation within 24 h [24], which indicates that the reduced motility of breast cancer cells with DACH1 overexpression was not due to inhibiting the cell proliferation. These results showed that DACH1 might inhibit the metastasis of breast cancer cells.

### DACH1 down-regulates the transcriptional activity of *MMP9* promoter

**Fig. 2:**
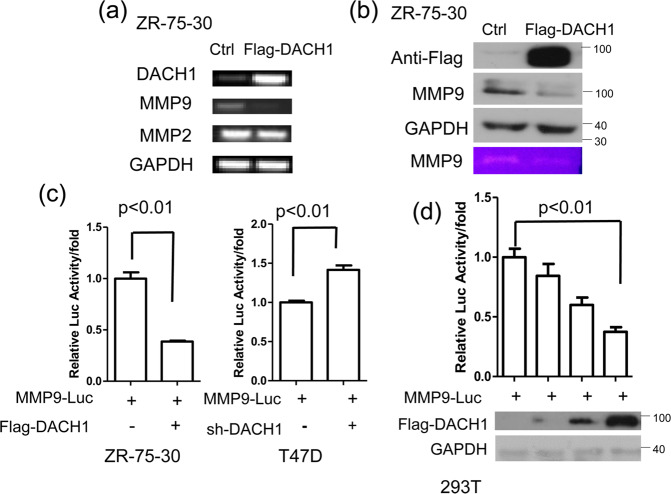
DACH1 down-regulates the level of MMP9. **a** ZR-75-30 cells were transfected with Flag-DACH1 or control vector, and then subjected to the analysis of *MMP9* and *MMP2* expression by RT-PCR. **b** Western blot assay evaluating the protein level of MMP9 in ZR-75-30 cells transfected with Flag-DACH1 or control vector (the first three lines). The medium from this assay was subjected to gelatin zymography (the last line). **c** Breast cancer cells were transfected with MMP-Luc together with or without Flag-DACH1 (left) or sh-DACH1 (right). Luciferase activity was measured 24 h after transfection. **d** HEK293T cells were transfected with MMP-Luc along with 0, 200, 400, 600 ng Flag-DACH1, and the luciferase activity was measured 24 h after transfection. There were three independent experiments with three parallel wells each. The data are representative of three independent experiments. Data are presented as means ± SD (*P* < 0.05, significant; ns, not significant).

The degradation of ECM is necessary for the invasion and metastasis of breast cancer. MMP9 and MMP2 exert an important effect on degrading the type IV collagen. DACH1 could decrease the expression of *MMP9* and *MMP2* in in gastric cancer [25], so the effect of DACH1 on the expression of *MMP9* and *MMP2* was examined in breast cancer cells. Reverse transcription-PCR (RT-PCR) assay showed that DACH1 overexpression reduced the mRNA level of *MMP9*, while had no noticeable variations in the expression of *MMP2* (Fig. 2a). MMP9 protein abundance was also reduced due to the overexpression of DACH1 in ZR-75-30 cells (Fig. 2b, the first three lines). At the same time, to examine the effect of DACH1 on the activity of MMP9 secreted by ZR-75-30 cells, the medium was subjected to the gelatin zymography assay, and the result showed that MMP9 activity was reduced with DACH1 overexpression (Fig. 2b, the last line). Luciferase reporter assay was carried out to explore the effect of DACH1 on the transcriptional activity of *MMP9* promoter. The overexpression of DACH1 decreased the activity of MMP9-driven reporter luciferase, while DACH1 knockdown promoted the transcriptional activity of *MMP9* (Fig. 2c). In addition, the reduction of reporter activity of *MMP9* promoter caused by DACH1 overexpression occurred in a dosage-dependent manner (Fig. 2d). Our data showed that DACH1 might inhibit the expression of MMP9 by reducing the transcriptional level of *MMP9*.

### DACH1 response element was identified in *MMP9* promoter

**Fig. 3:**
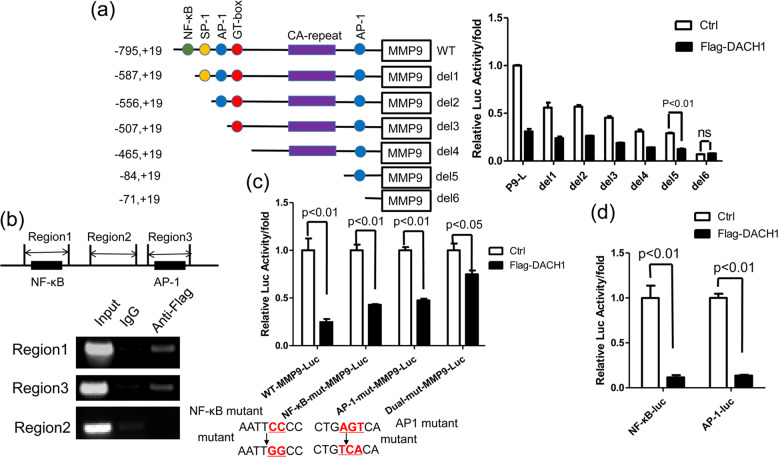
Determination of DACH1 binding sites in MMP9 promoter. **a** HEK 293 T cells were transfected with a series of 5’-deletion constructs of human MMP9 promoter-reporter plasmids together with or without Flag-DACH1. After 24 h, the luciferase activities were measured. The bar graph shows the fold changes in relative luciferase activity normalized against the β-galactosidase activity. There were three independent experiments with three parallel wells each. **b** ChIP assay showing the binding region of DACH1 in *MMP9* promoter. Up, PCR amplified region of *MMP9* promoter. Down, HEK 293 T cells were transfected with Flag-DACH1. Cross-linked chromatin was extracted from the cells and subjected to immunoprecipitation with anti-Flag antibody. The DNA regions were amplified by PCR. IgG was used as a negative control. **c** HEK 293 T cells were transfected with WT-MMP9-luc, NF-κB mut-MMP9-luc, AP-1 mut-MMP9-luc or dual-mut-MMP9-luc together with or without Flag-DACH1. Luciferase activity was measured 24 h after transfection. HEK 293 T cells were transfected with pNF-κB-Luc or pAP-1-luc along with or without Flag-DACH1. Luciferase activity was measured 24 h after transfection. Reporter-gene assay evaluating the regulation of site-specific mutational MMP9 luciferase reporter activity by DACH1 in HEK 293 T cells. The mutation of NF-κB: 5’-AATTCCCC-3’ to 5’-AATTGGCC-3’; the mutation of AP-1: 5’-CTGAGTCA-3’ to 5’-CTGTCACA-3’. **d** The luciferase activities of pNF-κB-Luc and pAP-1-luc were determined after transfected with Flag-DACH1 or control vector in HEK 293 T cells. There were three independent experiments with three parallel wells each. The data are representative of three independent experiments. Data are presented as means ± SD (*P* < 0.05, significant; ns, not significant).

To identify the response element of DACH1 in the *MMP9* promoter, six truncated *MMP9* promoters were constructed and cloned into the pGL3-Luc vector. The luciferase reporter assay showed that the full-length promoter (−795, +19) lost the most reporter activity with DACH1 overexpression compared with the control group, while the inhibitory effect of DACH1 on the reporter activity of *MMP9* promoter deletion (from del 1 to del 5) was considerably lessened compared with the full-length promoter (Fig. 3a). However, further deletion up to −71 (del 6) resulted in no significant changes in DACH1 inhibitory activity (Fig. 3a). It was noteworthy that DACH1 hardly had any inhibitory effect on the *MMP9* reporter activity with the deletion of nucleotides −84 to −72 (Fig. 3a). These data indicated that DACH1 might exert the repressive effects on the *MMP9* reporter activity mainly dependent on both the regions from nucleotides −795 to −588 and −84 to −72 in the *MMP9* promoter. The ChIP assay was performed to validate whether DACH1 could inhibit the activity of *MMP9* promoter through these two regions. Three primer pairs were used in the ChIP assay, including nucleotides −691 to −540 (region 1), −393 to 144(region 2) and −166 to −16 (region 3) of *MMP9* promoter. Among them, region 1 contained the NF-κB binding site, and region 3 contained the AP-1 binding site (Fig. 3b, top). DACH1 could associate with region 1 and region 3 (Fig. 3b, bottom), which is coherent with the results of the *MMP9* reporter assay, and these two regions contain NF-κB binding site and AP-1 binding site respectively. To further verify whether DACH1 could repress *MMP9* promoter through NF-κB and AP-1 binding sites, the full-length *MMP9* promoter that contained single mutation(NF-κB or AP-1 binding site mutant) or two site mutations(both NF-κB and AP-1 binding site mutant) were used in the reporter assay. The result showed that the repressive effect of DACH1 on the NF-κB or AP-1 mutant-Luc was lower than that on the WT-MMP9-Luc. As for the effect of DACH1 on the dual mutant-Luc, the effect was further reduced compared with the two single mutant-MMP9-luc (Fig. 3c). Besides, DACH1 significantly inhibited the activity of the NF-κB and AP-1 reporter, respectively (Fig. 3d). In summary, these results suggested that DACH1 might inhibit MMP9 expression by binding to the NF-κB and AP-1 response elements in the *MMP9* promoter.

### DACH1 interacts with p65 and c-Jun respectively

**Fig. 4:**
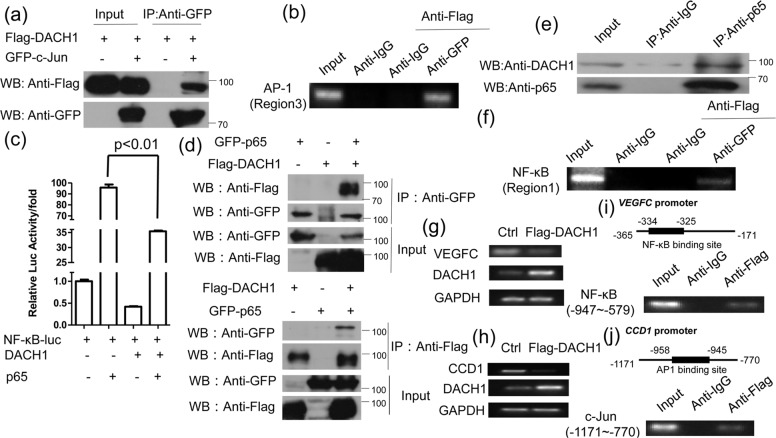
DACH1 interacts with p65 and c-Jun, respectively. **a** HEK 293 T cells transfected with Flag-DACH1 alone or both Flag-DACH1 and GFP-c-Jun were subjected to immunoprecipitation with anti-GFP antibody followed by Western blot with anti-Flag antibody. **b** HEK 293 T cells were transfected with Flag-DACH1 and GFP-c-Jun. After 24 h of transfection, DNA-protein complex were immunoprecipitated with anti-IgG or anti-GFP antibody, followed by re-immunoprecipitation with anti-Flag antibody. AP-1 binding site (region 3) was amplified by PCR. **c** HEK 293 T cells were transfected with pNF-κB-Luc and either DACH1 or p65, DACH1 and p65. Luciferase activity was measured 24 h after transfection. **d** HEK 293 T cells transfected with Flag-DACH1 and GFP-p65 were subjected to immunoprecipitation with anti-GFP antibody followed by Western blot with anti-Flag antibody or vice versa. **e** ZR-75-30 cells were subjected to immunoprecipitation with anti-p65 or anti-IgG antibody followed by Western blot with anti-DACH1 antibody. **f** HEK 293 T cells were transfected with Flag-DACH1 and GFP-p65. After 24 h of transfection, DNA-protein complex were immunoprecipitated with anti-IgG or anti-GFP antibody, followed by re-immunoprecipitation with anti-Flag antibody. NF-κB binding site (region 1) was amplified by PCR. There were three independent experiments with three parallel wells each. The data are representative of three independent experiments. Data are presented as means ± SD (P < 0.05, significant; ns, not significant). **g**, **h** ZR-75-30 cells were transfected with or without Flag-DACH1, and then subjected to the analysis of *CCD1* or *VEGFC* expression by RT-PCR. **i**, **j** Up, the schematic structure of NF-κB binding site in *VEGFC* promoter or AP1 binding site in *CCD1* promoter. Down, ZR-75-30 cells were transfected with Flag-DACH1. Cross-linked chromatin was extracted from the cells and subjected to immunoprecipitation with anti-Flag or IgG antibody. The DNA regions were amplified by PCR. IgG was used as a negative control.

Activator protein-1(AP-1) is an essential type of nuclear transcriptional activator, which is consists of the families of Jun, Fos, ATF, MAF and CREB [26]. They can bind to the target DNA sequence in the form of a homologous or heterologous dimer, and the typical AP-1 is a heterodimer composed of two subunits, c-Jun and c-Fos [27]. DACH1 could interact with c-Jun and repress the activity of AP-1 [23]. Our result also showed that DACH1 could interact with c-Jun in HEK 293 T cell lines (Fig. 4a), which is coincident to the previous study. A ChIP-reChIP assay was then performed to determine whether DACH1 could interact with c-Jun at the AP-1 binding site in the *MMP9* promoter. The result showed that DACH1 and c-Jun could bind to the AP-1 binding site (region 3) (Fig. 4b). Our data suggested that DACH1-c-Jun complex might bind to AP-1 binding site in *MMP9* promoter.

Next, we examined whether NF-κB p65 was involved in the repressive effect of DACH1 on the transcription of *MMP9* via the NF-κB binding site. The luciferase reporter data showed that overexpression of p65 could promote the activity of NF-κB-luc compared with the control, while the activity of NF-kB-luc was reduced in the present of DACH1 and p65 compared with p65 overexpression only. This result suggested that DACH1 could down-regulate the activity of *MMP9* promoter by inhibiting the transcriptional activity of p65 (Fig. 4c). CoIP assay showed that DACH1 could associate with p65 (Fig. 4d), and the same immunoprecipitation experiment was carried out using endogenous DACH1 and p65 (Fig. 4e). The ChIP-ReChIP experiment showed that DACH1 and p65 could bind to the NF-κB binding site (region 1) in the form of a complex (Fig. 4f). These data indicated that DACH1 might interact with p65 and c-Jun, at the NF-κB and AP-1 binding sites respectively, and then inhibit the activity of the *MMP9* promoter.

*VEGFC* and *CCD1* are target genes of NF-κB and C-jun [28, 29], respectively, so we studied the effect of DACH1 on the mRNA level of these two genes. DACH1 overexpression down-regulated the mRNA level of *CCD1* and *VEGFC* (Fig. 4g and h). Then ChIP assay was performed to examine whether DACH1 repressed the transcription level of *CCD1* and *VEGFC* via binding to the promoter of these two genes. The result showed that DACH1 could bind to the NF-κB binding site in the promoter of *VEGFC* and AP-1 binding site in the promoter of *CCD1* (Fig. 4i and j). These data showed that DACH1 could regulate the transcription of the target genes of NF-κB or C-jun by binding to the NF-κB binding site or AP-1 binding site.

### DACH1 recruits HDAC1 to the NF-kB binding site

**Fig. 5:**
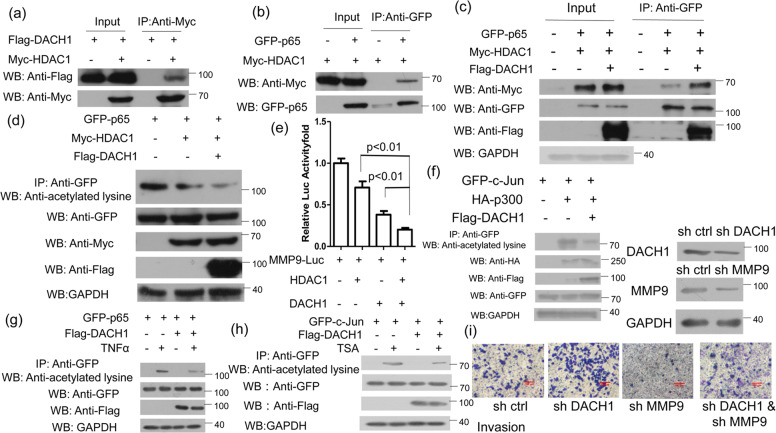
DACH1 recruits HDAC1 to the NF-kB binding site. **a** HEK 293 T cells transfected with Flag-DACH1 alone or Flag-DACH1 and Myc-HDAC1 were subjected to immunoprecipitation with anti-Myc antibody followed by western blot with anti-Flag or anti-Myc antibody. **b** HEK 293 T cells transfected with GFP-p65 alone or together with Myc-HDAC1 were subjected to immunoprecipitation with anti-GFP antibody followed by Western blot with anti-Myc or anti-GFP antibody. **c** HEK 293 T cells transfected with GFP-p65 and Myc-HDAC1 along with or without Flag-DACH1 were immunoprecipitated with anti-GFP antibody followed by Western blot with anti-Myc, anti-GFP, anti-Flag antibody. **d** HEK 293 T cells transfected with the indicated plasmids were subjected to immunoprecipitation with anti-GFP antibody followed by Western blot with anti-acetylated lysine antibody. All cells were treated with TNFα (40 ng/ml) for 4 h before harvest. TNFα was used to activate the NF-κB signaling pathway and promote the acetylation level of p65. **e** HEK 293 T cells were transfected with MMP9-luc and either HDAC1 or DACH1, HDAC1 and DACH1. **f** HEK 293 T cells were transfected with the related plasmids for 24 h, and then the cell lysis were subjected to immunoprecipitation with anti-GFP antibody followed by western blot with anti-acetylated lysine antibody. **g** HEK 293 T cells were transfected with GFP-p65 along with or without Flag-DACH1. Cells were treated with or without TNFα (40 ng/ml) for 4 h before harvest. The lysis were subjected to immunoprecipitated with anti-GFP antibody followed by anti-acetylated lysine antibody. **h** HEK 293 T cells transfected with the related plasmids for 24 h and then the cells treated by TSA (1 μM) for 6 h before harvest were immunoprecipitated with anti-GFP antibody followed by western blot with anti-acetylated lysine antibody. **i** MCF7 cell invasion assays was performed in 24-well chambers with Matrigel. Cells were transfected with sh Ctrl, sh DACH1, sh MMP9 or sh DACH1 and sh MMMP9, and then inoculated into the upper chamber. After cultured 24 h, cells on the lower surface of the filter were stained and photographed. The scale bars stand for 100 μm. Luciferase activity was measured 24 h after transfection. Experiments were performed in triplicates and the data are representative of three independent experiments. The data are representative of three independent experiments. Data are presented as means ± SD (*P* < 0.05, significant).

Previous study has demonstrated that DACH1 can interact with c-Jun and promote the recruitment of histone deacetylase 1 (HDAC1) to AP-1 site, which then suppress the activity of *Cyclin D1* promoter [22]. DACH1 can also repress the transcription and transactivation of c-Jun by binding a corepressor complex, including HDAC1 [23]. So we examined whether the effect of the association of DACH1 and p65 on repressing the expression of *MMP9* was also dependent on the recruitment of HDAC1. The CoIP assay showed that both DACH1 and p65 could interact with HDAC1 (Fig. 5a, b), which was consistent with previous reports [22, 30]. Furthermore, the data showed that DACH1 overexpression promoted the interaction between p65 and HDAC1 (Fig. 5c). HDAC1 could interact with p65 and suppress the transcriptional activity of p65 via deacetylating p65 [30]. Similarly, HDAC1 reduced the acetylation level of p65, and the acetylation level of p65 was further reduced with co-overexpression of both HDAC1 and DACH1 (Fig. 5d). The luciferase assay showed that overexpression of HDAC1 or DACH1 alone could downregulate the activity of MMP9-Luc compared with the control, while co-transfection with HDAC1 and DACH1 further reduce the activity of MMP9-Luc compared with transfection of HDAC1 or DACH1 alone (Fig. 5e). Previous studies have demonstrated that acetylation of c-Jun lysine residues by the p300 took an important effect in the activation of keratin 16 gene expression [31]. So we tried to examine whether DACH1 overexpression could regulate the acetylated level of c-Jun. As shown in Fig. 5f, DACH1 overexpression inhibited the acetylation of c-Jun mediated by p300. The repressing effect of DACH1 on the p65 or c-Jun acetylation was further identified by treating cells with TNFα or TSA before harvest (Fig. 5g, h). It has been reported that HDAC3 knockdown could promote the acetylation of c-Jun [32], and TSA is the inhibitor of HDACs family. To examine whether the effect of DACH1 on the invasion of breast cancer cells dependent on MMP9, the transwell invasion assay was performed and the result revealed that *DACH1* knockdown upregulated the invasiveness of MCF7 cells, while *MMP9* knockdown inhibited the invasiveness of cells. Both *DACH1* and *MMP9* knockdown decrease the promoting role of DACH1 knockdown only in the invasion of MCF7 cells (Fig. 5i). However, MCF7 cells transfected with both sh DACH1 and sh MMP9 were still characterized by greater invasion ability compared with cells transfected with sh MMP9 only. This data demonstrated that DACH1 mainly repress the invasion of breast cancer cells via MMP9 pathway. These results showed that DACH1 might enhance the interaction between HDAC1 and p65 and reduce the acetylation level of p65, thereby suppressing the transcriptional activity of p65 and down-regulating the expression of *MMP9*.

### DACH1 inhibits breast cancer metastasis in vivo

**Fig. 6:**
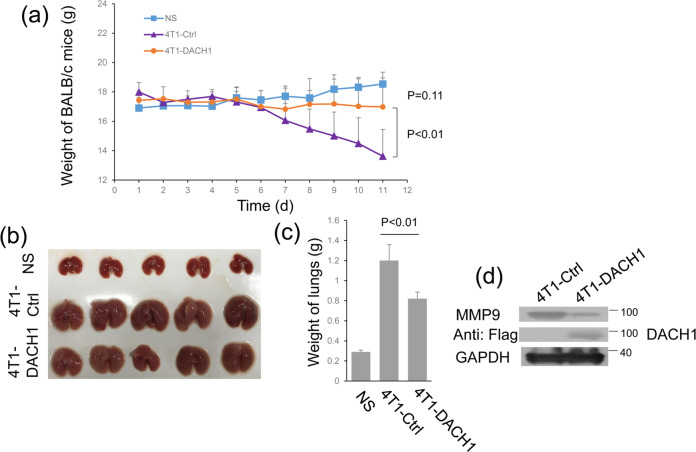
DACH1 modulates the metastasis of 4T1 cells in BALB/c mice. **a** Line graph image showing the effect of DACH1 overexpression on the weight of BALB/c mice. **b** The images showing the effect of DACH1 overexpression on lung size in mice. **c** The bar graph shows the quantitative measure of the weight of lung in mice. **d** Western blots showing the MMP9 expression when overexpressed DACH1 into 4T1 cells. The data are representative of three independent experiments. Data are presented as means ± SD (*P* < 0.05, significant).

To study the role of DACH1 in metastasis in vivo, an animal metastasis model was used, where BALB/c mice were injected with 4T1 cells [33]. Mice injected with 4T1 cells transfected with control vector exhibited significant weight loss when compared to the other two groups (Fig. 6a). The sizes and weights of the lungs of BALB/c mice that overexpressed DACH1 were also significantly decreased compared with 4T1-Ctrl group, but were still bigger than those of NS group (Fig. 6b, c). Furthermore, overexpression DACH1 in 4T1 cells can drastically reduce the MMP9 expression in cells through western blot (Fig. 6d). These results demonstrate that DACH1 represses breast cancer metastasis in an animal metastasis model.

## Discussion

*Dachshund* (*dac*) encoding a novel nuclear protein was first identified in Drosophila in 1994, it was essential for the development of the eyes [34]. DACH is a vital member of the Retinal Determination Gene Network (RDGN), which is the primary regulatory pathway for the Drosophila melanogaster eye specification [35, 36]. Recent studies have demonstrated that RDGN members (DACH, EYA, SIX, etc) are associated with the initiation and progression of cancer. *DACH1* is a human homolog of the *Dachshund* gene, and it has been reported to there is an abnormal expression in a variety of carcinomas, such as breast cancer [37], gastric cancer [38], renal Cell Carcinoma [39] and others. DACH1 is generally considered as an inhibitor of cancer cell invasion and metastasis, while the molecule mechanism in the tumor cell migration is not entirely understood. Our study showed that DACH1 could repress the breast cancer cell metastasis and invasion by inhibiting the expression of *MMP9* at the transcriptional level. Mechanistically, DACH1 could interact with p65 and c-Jun at AP-1 and NF-κΒ binding sites respectively in the promoter of *MMP9*. DACH1 could upregulate the association of HDAC1 and p65 and promote the deacetylation of p65, thereby repressing the transcriptional activity of p65 and *MMP9* expression. Our work showed that DACH1 exerted an important effect on inhibiting the metastasis of breast cancer cells, and further studies would provide a novel strategy for treating breast cancer invasion and metastasis in humans.

DACH1 can repress the transcription of *interleukin-8* gene through AP-1 and NF-κΒ binding sites [16, 40]. DACH1 could also inhibit *MMP9* expression via both AP-1 (−79 to −73) and NF-κΒ (−600 to −590 bp) binding sites (Fig. 3b and c). It should be noted that the inhibitory effect of DACH1 was not completely eliminated in the presence of dual-mut-MMP9-Luc (both AP-1 and NF-κΒ binding sites mutant) (Fig. 3c). We speculated that there were two possibilities. On one hand, the *MMP9* promoter sequences spanning −795 to +19 is considered as the major regulatory region, including one NF-κB binding site and two AP-1 binding sites. These sites play crucial roles in the regulation of *MMP9* expression. As for the two AP-1 binding sites, one is called proximal AP-1 binding site (from −79 to −73 bp) and the other is distal AP-1 binding site (from −531 to −527 bp). The previous data showed that the proximal AP-1 binding site have more critical effect on the regulation of *MMP9* expression compared with the distal AP-1 binding site [11]. In our study, we showed that DACH1 could repress the activity of MMP9-Luci through the NF-κB and proximal AP-1 binding sites (Fig. 3a–c). However, we could not completely rule out that DACH1 might also inhibit the transcription of *MMP9* via the distal AP-1 binding site. On the other hand, a sequence analysis showed that there are two DACH1 binding sites (5’-CAGCTG-3’, derived from the JASPAR^2020^ Database) in the *MMP9* promoter (−795, +19) used in our study. One binding site is localized in −755~−746, and the other one is localized in −12~−3. We also performed the ChIP experiment to examine whether p65 affect the binding of DACH1 to the promoter of *MMP9*. The result showed that the binding of DACH1 to the promoter of *MMP9* (NF-κB binding site) was almost undetectable with p65 knockdown (data not shown). The binding site of p65 to *MMP9* promoter is very close to the potential binding site (−755~−746) of DACH1 to the promoter of *MMP9*. Therefore, DACH1 might not bind to *MMP9* promoter through this binding site (−755~−746), but this needs to be further studied. In addition, the role of DACH1 in the regulation of *MMP9* promoter used in this research via another binding site (−12~−3) was also excluded by the luciferase assay. As shown in Fig. 3a, DACH1 overexpression had no effect in the activity of MMP9-Luc (del6) compared with the control group. According to our analysis and experimental data, it is unlikely that DACH1 repressed the transcription of *MMP9* via binding to the promoter of *MMP9* directly. So we speculated that DACH1 might repress the transcription of *MMP9* via distal AP-1 binding site mediated by c-Jun or other unknown transcriptional factors might regulate the transcription of *MMP9*, and then DACH1 could affect the expression of *MMP9* via these unknown transcriptional factors. However, these predictions need to be further identified.

DACH1 could interact with c-Jun at the proximal AP-1 binding site of *MMP9* promoter (Fig. 4b), and then suppressed *MMP9* expression. It has been reported that DACH1 interacts with c-Jun by the Box-N domain of DACH1 and δ domain of c-Jun, [23] and then represses the expression of c-Jun target gene *cyclin A* by recruiting HDAC1 [41]. Also, DACH1 repressed the actylation of c-Jun mediated by p300 (Fig. 5f). So we speculate that DACH1 inhibits the expression of *MMP9* by interacting with c-Jun and promoting to erase the acetylation of c-Jun.

In the classical NF-κB signaling pathway, p65/p50 complex binds to the NF-κB binding site of the target gene promoter and activate the transcription of the target gene [12, 13]. p65 is the catalytic core which can initiate the transcription via its C-terminal transactivation domains (TADs) [42]. In our research, DACH1 could interact with p65 at the NF-κB binding site in the *MMP9* promoter (Fig. 4b). Although p65 and p50 usually work as a dimer, whether there is any interaction between DACH1 and p50 is uncertain. Our data showed that DACH1 decreased the acetylation level of p65 by recruiting HDAC1, and then inhibit the expression of *MMP9*. Besides HDAC1, DACH1 can also inhibit the expression of target genes via recruiting HDAC3 and NCoR [43, 44]. Whether these co-factors also participate in the regulation of *MMP9* expression is still unclear, and it deserves further studies.

In conclusion, our data have shown that DACH1 inhibited breast cancer metastasis by down-regulating the expression of *MMP9*. In terms of mechanism, DACH1 interacted with p65 and c-Jun at the NF-κB and AP-1 binding sites respectively. Moreover, DACH1 reduced the acetylation level of p65 through recruiting HDAC1, and then repressed the transcriptional activity of p65. Our results confirmed the effect of DACH1 on the inhibition of breast cancer metastasis. And this new mechanism of the inhibition of breast cancer metastasis mediated by DACH1 may provide a novel insight into the treatment of breast cancer metastasis.

## Materials and methods

### Cell culture and transfection

HEK-293T, MCF-7, ZR-75-30 and T47D cells have been used in our previous study and were cultured as previously described [45]. Cells were grown at 37 °C in a humidified 5% CO_2_ atmosphere and transfected plasmids using Lipofectamine 2000 (Invitrogen, Auckland, New Zealand) according to the manufacturer’s specifications.

### Plasmids and antibodies

The pKW-Flag-DACH1 plasmid was a gift from Dr Richard G. Pestell (Thomas Jefferson University, USA). Human MMP9 promoter from −795 to +19 was cloned from ZR-75-30 cells using the primers: forward, 5’-ggggtacctttagggacaaagagcccc-3’ (including KpnI site), reverse, 5’-ccgctcgaggtgagggcagaggtgtc-3’ (including XhoI site), and inserted into pGL3 vector. Six truncations of MMP9 promoter were produced using the same method, except the template and primers. The template was pMMP9-Luc (−795, +19), and the forward primers of del1~del5 were as previously described [45], the forward primers of del6: 5’-ggggtacccacttgcctgtcaaggagg-3’, the reverse primer were all used 5’-ccgctcgaggtgagggcagaggtgtc-3’. pcDNA3.1-JUN and pGL3-AP1-Luc were gifts from Dr. D. Wang (Tsinghua University, China), Using pcDNA3.1-JUN as a template to generate pGFP-c-Jun, the primers were as followed: forward, 5’-ggaattccatgactgcaaagatggaaacg-3’, reverse, 5’-cgcggatcctcaaaatgtttgcaactgct-3’. pNF-κB-Luc, pMyc-HDAC1, sh-DACH1 and pGFP-p65 were acquired as previously described [45–47].

Mouse anti-β-actin (sc-47778, 1:1000 for WB) was obtained from Santa Cruz Biotechnology; Rabbit acetylated lysine antibody was obtained from CST (9441, 1:2000 for WB); Mouse anti GAPDH was purchased from ORIGENE (TA802519, 1:6000 for WB); Mouse anti-Flag® M2 (F1804, 1:3000 for WB), Rabbit anti-Flag (F7425, 1:500 for IP) and rabbit anti-MYC (PLA0001, 1:500 for IP; 1:3000 for WB) antibodies were purchased from Sigma-Aldrich; Rabbit anti-GFP (GTX113617, 1:500 for IP; 1:3000 for WB) and anti-HA (GTX115044, 1:4000 for WB) were obtained from GeneTex. Rabbit p65 (ab16502, 1:500 for IP; 1:5000 for WB) and DACH1 (ab226176, 1:4000 for WB) antibodies were obtained from Abcam; Rabbit anti-MMP9 antibodies were purchased from Wanlei (WL02141, 1:500 for WB).

### Transwell and scratch wound-healing assays

Transwell and scratch wound-healing assays were executed as previously described [45]. Cells were transfected with appropriate plasmids. 24 h after transfection, the cells were counted using cell-count boards. In the scratch wound-healing assay, ZR-75-30 or MCF-7 cells were seeded in 35-mm culture dish at a density of 3 × 10^5^ cells, and after 12 h incision of the wounds were performed by scratching the cell layers with a 200 μl pipette tip. Cells with the same field of view were photographed under phase-contrast microscopy immediately and 24 h after the scratching. In the Transwell assay, pre-coated (invasive) or without (migration) chambers Matrigel (BD Transduction, Franklin Lake, NJ) were placed in a 24-well plate, 1 × 10^4^ ZR-75-30 cells in 100 μl serum-free medium were transferred into the upper chamber and the lower chambers were filled with 500 μl RPMI-1640 medium containing 10% fetal bovine serum. After incubation for 24 h, migrating cells stained with 0.5% crystal violet solution were photographed.

### Luciferase reporter assay

Promoter activity was determined by a luciferase assay system. Cells were seeded at a density of 1 × 10^5^ cells into a 24-well plate. After 24 h, the cells were transfected with pertinent plasmids using Lipofectamine 2000 according to the company’s specification. After cultured 24 h, according to the manufacturer’s instructions, the cells were used to carry out luciferase and Renilla activity assays (Promega, Madison, WI, USA). β-galactosidase reporters as an internal control.

### RNA extraction and RT‑PCR

Total RNA was retrieved from ZR-75-30 cells using RNAiso Reagent (Takara, Dalian, China). Total RNA (3 μg) was reverse transcribed using oligo (dT) primer and a Reverse Transcription System (Takara). The single-stranded cDNA was amplified by PCR using specific primers:

*MMP9* (forward) 5’-tgtaccgctatggttacac-3’

*MMP9* (reverse) 5’-ccgcgacaccaaactggat-3’

*MMP2* (forward) 5’-gtgaagtatggcaacgccga-3’

*MMP2* (reverse) 5’-cggtcgtagtcctcagtggt-3’

*DACH1* (forward) 5’-gaatagagccatagttcaaaagagg-3’

*DACH1* (reverse) 5’-agtcatttaagaccctgagactatc-3’

*CCD1* (forward) 5’- gctgctcctggtgaacaagc-3’

*CCD1* (forward) 5’- aagtgttcaatgaaatcgtgcg-3’

*VEGFC* (forward) 5’- ttatgcaagcaaagatctgg-3’

*VEGFC* (forward) 5’- cgtggcatgcattgagtctt-3’

*GAPDH* (forward) 5′-gggttgaaccatgagaagt-3′

*GAPDH* (reverse) 5′-gactgtggtcatgagtcct-3′

### Western blot, CoIP assays

Western blot and co-immunoprecipitation (CoIP) assays were conducted as previously described [48, 49]. Total cellular proteins were extracted using RIPA buffer. The cell lysates were clarified and incubated with the appropriate antibodies for 6 h at 4 °C, followed by incubation with pre-cleared Protein A/G agarose beads (Santa Cruz, #sc-2003) for 4 h at 4 °C. Proteins of immunoprecipitates and total cell lysates were separated by 8% SDS-PAGE, transferred onto a PVDF membrane, blocked with 5% milk in PBST and analyzed by immunoblotting with primary and secondary antibodies.

### ChIP

Chromatin immunoprecipitation (ChIP) assays were carried out as previously described [50]. HEK-293T cells were treated with 1% formaldehyde to cross-link proteins with DNA on a 100-mm culture dish. The cells were washed with ice-cold PBS, lysed in warm 1% SDS lysis buffer, and incubated for 20 min at 4 °C. Sonication was applied to the cell debris to shear DNA to lengths between 200 and 1000 bp. Equal amounts of chromatin supernatants, containing anti-Flag antibody or the same amount of control IgG, were incubated overnight at 4 °C with shaking. To obtain ChIP-reChIP, the chromatin immunocomplexes were further incubated with an anti-GFP antibody. Chromatin solutions were precipitated 2 h with agitation at 4 °C using 30 μl of pre-cleared Protein A/G agarose beads. The precipitated DNAs were analyzed by PCR.

The primers used in the ChIP PCR analysis for *MMP9* promoter were as follows:

Region 1 (forward) 5’-gccatgtctgctgttttctagagg-3’

Region 1 (reverse) 5’-ttcctctccctgcttcatctgg-3’

Region 2 (forward) 5’-ctcagggagtcttccatcactttc-3’

Region 2 (reverse) 5’-agcatgagaaagggcttacaccac-3’;

Region 3 (forward) 5’-tggtgtaagccctttctcatgc-3’

Region 3 (reverse) 5’-gttgtgggggctttaaggaggc-3’

The primers used in the ChIP PCR analysis for *CCD1* promoter were as follows:

Forword 5’-tcagatcagtacactcgttt-3’

Reverse 5’-ggagactcttcgggctgcct-3’

The primers used in the ChIP PCR analysis for *VEGFC* promoter were as follows:

Forword 5’-acaagaactcgggagtggcc-3'

Reverse 5’-atctctccctccccgcccct-3’

### Gelatin zymography

Gelatin zymography assays were conducted as previously described [45]. According to MMP Zymography Assay Kit (XF-P17750). ZR-75-30 cells were transfected with appropriate plasmids. After 24 h, the cells were washed and incubated in serum-free medium for 24 h. Culture supernatants were collected and electrophoresed on gelatin containing 8% SDS-polyacrylamide gel. After treated in the buffer A followed by incubation in buffer B at 37 °C overnight, the gel was stained and destained.

### Mice metastasis model

Female BALB/C nude mice (5–6-weeks old, 16–18 g) were obtained from Liaoning Changsheng Biotechnology and maintained under specific pathogen-free (SPF) conditions. All experiments were carried out according to the regulation set by the Ethics Committee for Biology and Medical Science of Dalian University of Technology. Simple random sampling method was used for the group allocation of experimental mice and the detection was performed blindly.15 mice were divided into three groups randomly. 10^7^ 4T1 cells suspended in normal saline was injected into the tail vein of the BALB/c mice. 4T1-Ctrl group was injected with 4T1 cells stably transfected with pcDNA3.1-3×Flag vector. 4T1-DACH1 group was injected with 4T1 cells stably transfected with pcDNA3.1-3×Flag-DACH1. NS group was injected with 100 μl normal saline only. After 11 days, the animals were killed under an anesthesia and the lungs were removed surgically for the assay.

### Statistical analysis

All blots were derived from the same experiment and were processed in parallel. Data were presented as mean ± SDs and Student’s *t*-test (unpaired, two-tailed) was used to compare two groups of independent samples. All the experiments were repeated at least three times. Statistical significance was considered at the *p* < 0.05 level.

## Data Availability

The data supporting the findings of this study are available from the corresponding author upon reasonable request.
